# Characteristics of Adverse Events Following Immunization Reporting in Children: The Japanese Adverse Drug Event Report Database

**DOI:** 10.3390/vaccines8030357

**Published:** 2020-07-03

**Authors:** Aoi Noda, Takamasa Sakai, Masami Tsuchiya, Gen Oyanagi, Taku Obara, Nariyasu Mano

**Affiliations:** 1 Division of Preventive Medicine and Epidemiology, Tohoku University Tohoku Medical Megabank Organization, Sendai, Miyagi 980-8573, Japan; a.noda@megabank.tohoku.ac.jp; 2 Division of Molecular Epidemiology, Environment and Genome Research Center, Graduate School of Medicine, Tohoku University, Sendai, Miyagi 980-8573, Japan; 3 Department of Pharmaceutical Sciences, Tohoku University Hospital, Sendai, Miyagi 980-8574, Japan; gen.oyanagi@hosp.tohoku.ac.jp (G.O.); mano@hosp.tohoku.ac.jp (N.M.); 4 Drug Informatics, Faculty of Pharmacy, Meijo University, Nagoya, Aichi 468-8503, Japan; tksakai@meijo-u.ac.jp; 5 Laboratory of Clinical Pharmacy, Tohoku University Graduate School of Pharmaceutical Sciences, Sendai, Miyagi 980-8574, Japan; masami-tuchiya@miyagi-pho.jp; 6 Department of Pharmacy, Miyagi Cancer Center, Natori 981-1293, Miyagi, Japan

**Keywords:** adverse events following immunization, vaccine, children, spontaneous reports

## Abstract

The present study aimed to describe the trends and characteristics of adverse events following immunization (AEFI) reporting for children in the Japanese Adverse Drug Event Report database (JADER). We used 6280 AEFI reports for children aged <19 years among 504,407 ADR reports included in the JADER from 2004 to 2017. The number of AEFI reports gradually increased among children aged <10 years and was the highest in 2011 among children aged 10–19 years. The number of suspected vaccines per AEFI report increased after 2011 among children aged <10 years. The percentage of “death” and “did not recover” as AEFI outcomes reported were 4.3% and 3.7% among children aged <10 years and 0.2% and 21.1% among children aged 10–19 years, respectively. The most frequently reported vaccine–reaction pair was *Haemophilus influenzae* type b conjugate vaccine and pyrexia among children aged <10 years and recombinant adsorbed bivalent human papillomavirus-like particle vaccine and a loss of consciousness among children aged 10–19 years. It is necessary to consider the Weber effects to understand the trend and characteristics of AEFI reporting because pharmacovigilance activity regarding vaccination is not sufficient in Japan.

## 1. Introduction

Vaccines are the most widely used medicine in children worldwide. They are usually administered not only to patients but also to healthy individuals, and serious adverse events following immunization (AEFI) rarely occur. Because there are many types of vaccines, including combination and live vaccines, the appearances and types of adverse reactions are diverse, especially in children. Therefore, the monitoring of AEFI is important [1], and, specifically, a well-functioning spontaneous reporting system of AEFI is one of the essential tools for the evaluation of post-marketing surveillance for vaccine safety [2]. Several studies have also been carried out to describe the trends and characteristics of adverse drug reaction (ADR) reports, including vaccine reports, to evaluate the quantity and quality of suspected ADR data among children [3,4,5,6]. Hesitancy concerning vaccines because of vaccine safety skepticism is prevalent in Japan [7,8]. For example, the human papillomavirus vaccine in Japan has had a different situation from other countries, and the proactive recommendation for the human papillomavirus vaccine has still been suspended. The success of national immunization program needs the public’s confidence in vaccines, by monitoring the risk–benefit balance of vaccines and providing relevant information through pharmacovigilance activities [9]. Therefore, it is necessary to describe the trends and characteristics of AEFI reporting in the Japanese spontaneous reporting system to understand what is happening regarding vaccines.

In 2011, the WHO established the Global Vaccine Safety Blueprint (GVSB) as a strategic document for ensuring vaccine safety [10]. The GVSB has aimed that all countries have the minimum pharmacovigilance system necessary to ensure vaccine safety. However, in Japan, the history of individual vaccinations has not been recorded in a database and the rate of AEFI cannot be calculated. The Ministry of Health, Labour and Welfare (MHLW) has collected AEFI since 1948, owing to the Preventive Vaccination Law, and since 1967, because of the Pharmaceutical Affairs Law. Since 2012, the Pharmaceutical and Medical Devices Agency (PMDA), which was established by the MHLW, has been made part of ADR reports available to the public through the Japanese Adverse Drug Event Report database (JADER). The JADER includes ADR reports after 2004 and features details such as patient demographics, drug information, adverse events, and disease data (http://www.pmda.go.jp/safety/reports/hcp/0001.html). Legislation dealing with vaccines and nonvaccines has differed, but the amendment of the Preventive Vaccination Law in 2013 made it a legal duty to report AEFI, and the PMDA began collecting these reports unitarily in the same way as ADR reports concerning nonvaccines are collected. Accordingly, the JADER is the only way to monitor the reporting of AEFI in Japan.

The purpose of the present study was to describe the trends and characteristics of AEFI reports in children.

## 2. Materials and Methods

Data, which were included in the JADER from 1 April 2004, to 31 December 2017, were extracted and downloaded in April 2018. These reports were checked and evaluated for whether or not the AEFI report was serious before being registered in the JADER by the PMDA, and the JADER in principle comprises serious ADR reports selected by the PMDA. A single AEFI report often includes multiple AEFIs, which can include nonserious events, including pyrexia. Based on the International Conference on Harmonization of Technical Requirements for Registration of Pharmaceuticals for Human Use guideline E2B guidelines [11], adverse events were considered serious when they resulted in death, were life threatening, required hospitalization or the prolongation of existing hospitalization, resulted in persistent or significant disability or incapacity, were congenital abnormalities or birth defects or were any other medically significant events. According to the law called the “Pharmaceutical and Medical Device Act”, the PMDA recommends that pharmaceutical companies and healthcare professionals report AEFIs, even if the causal relationship between vaccine use and AEFI is unclear. As for pharmaceutical companies, they are required strictly to report all ADRs within the reporting deadline, differently from healthcare facilities and there is the punishment if they did not report an AEFI. The administrative penalty, such as a business-suspension order or orders to improve business operations, has been applied in Japan. Patients and consumers cannot report AEFIs directly through a system called the Drugs and Medical Devices Safety Information Reporting System. As for patients, the Direct Patient Reporting System for ADR, in which patients and consumers can report ADRs directly to the PMDA, was tentatively started from 2012 as a pilot program and a full-scale operation of the system was started on 26 March 2019.To make the system known to the patients and consumers, the PMDA requested to local government to enlighten the system through newsletters and websites; however, the number of reports was small and it might have not yet been fully disseminated. However, the JADER has not included the reports from this system yet. The reports that were submitted by consumers in the JADER were almost always the reports that come from pharmaceutical companies.

The JADER data consist of four elements: (1) patient demographic information, (2) drug information, (3) adverse events, and (4) diseases. We examined the trends and characteristics of these AEFI reports with respect to two age groups: children aged <10 and children aged 10–19 years. The adverse reaction and underlying disease fields in the JADER were based on the Japanese version of the *Medical Dictionary for Regulatory Activities* (MedDRA^®^/J) and were coded as “preferred terms” (PTs). We used MedDRA^®^/J version 21.0 (International Council for Harmonisation of Technical Requirements for Pharmaceuticals for Human Use, Geneva, Switzerland) in the present study. The information included patient details (age and sex), the type of report sender, the qualifications of the reporters, suspected drugs, outcomes resulting from ADR reports, and ADRs coded according to PTs. Age, sex, the type of report sender (pharmaceutical company or healthcare facility), the qualifications of the reporters (doctor, pharmacist, healthcare professional, consumer, or lawyer), the number of suspected drugs per ADR report, and outcomes from ADR reports (cured, recovering, did not recover, recovering with sequelae, death, or unexplained) were collected and were compared between children aged <10 and children aged 10–19 years by the chi-square test. Statistical analyses were conducted using SAS version 9.4 (SAS Institute, Inc., Cary, NC, USA). We extracted ADR reports for children aged <10 and 10–19 years. The 10 most frequently reported vaccines, reactions, and vaccine–reaction pairs were determined according to age (<10 or 10–19 years old). Time trends for the number of reports and the frequently reported vaccine, reaction, and drug–reaction pairs were also determined. Ethical approval for the study was obtained from the Institutional Review Board of Tohoku University School of Medicine (2017-1-506).

## 3. Results

### 3.1. Characteristics of the Reports in the JADER

**Figure 1:**
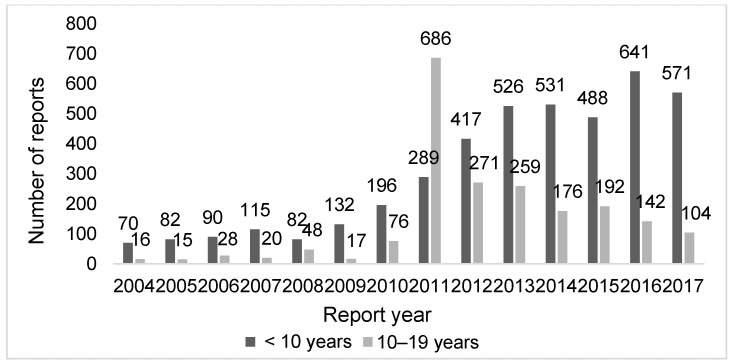
Annual AEFI reports in children in Japan for the period 2004–2017 according to age group. Abbreviation: AEFI, adverse event following immunization.

A total of 504,407 ADR reports from April 2004 to December 2017 were downloaded from the JADER in April 2018. Of these, there were 386,400 spontaneous reports (76.6%), of which 37,534 (7.4%) were reports with an unknown age of the patient. After the extraction of 27,800 ADR reports for children aged <10 and 10–19 years, we excluded ADR reports associated with nonvaccine (*n* = 21,434) and those without suspected vaccine (*n* = 86) reports. A total of 6280 (1.2%) reports were finally included in this analysis. Approximately 70% of the AEFI reports involved children aged <10. The percentages of AEFI reports corresponding to boys and girls were 53.7% and 42.6% among children aged <10, and 10.1% and 89.7% among children aged 10–19 years, respectively. The characteristics of AEFI reports were significantly different between age groups (all *p* < 0.0001). Regardless of age, approximately 99% were sent in from pharmaceutical companies. In some cases, multiple reporters were involved in a single report. Specifically, with respect to children aged 10–19 years, approximately 30% of the AEFI reports were submitted by consumers. The percentages of multiple-vaccine exposure reports among children aged <10 and 10–19 years were 37.8% and 1.1%, respectively (Table 1). The number of AEFI reports gradually increased among children aged <10 and was exceptionally high in 2011 among children aged 10–19 years (Figure 1). The number of suspected vaccines per AEFI report increased after 2011 among children aged <10.

### 3.2. Outcomes Associated with AEFI Reports

Among the 6280 reports analyzed, a total of 15,905 AEFIs were reported. The percentage of patients for whom the outcome was reported as “death” and “did not recover” was 4.3% and 3.7% among children aged <10 and 0.2% and 21.1% among children aged 10–19 years, respectively (Table 1).

### 3.3. Ten Most Frequently Reported Vaccines

**Figure 2:**
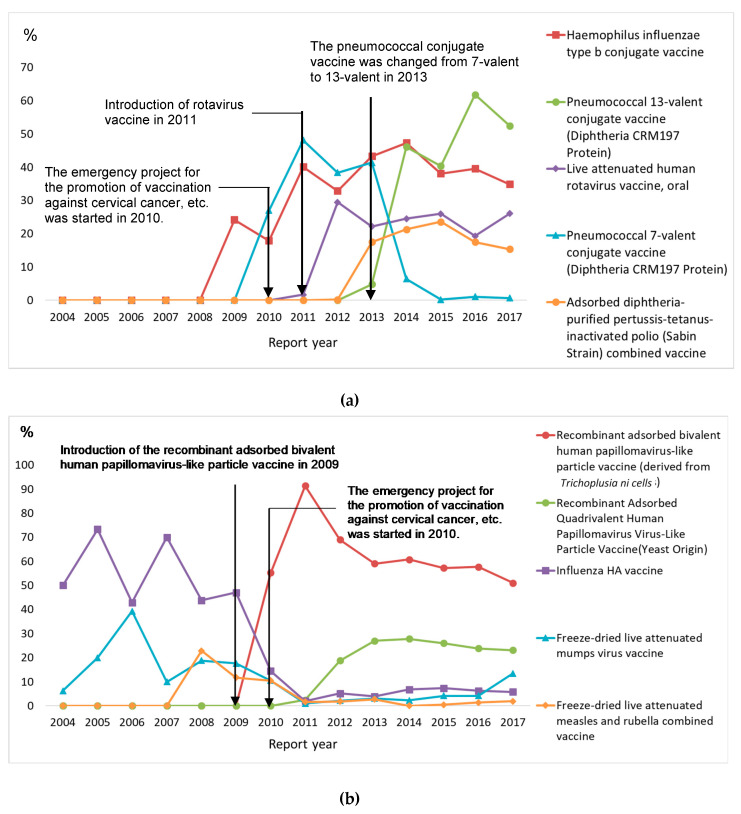
Time trend of the five most frequently reported vaccines according to age group. (**a**) Children <10 years old. (**b**) Children 10–19 years old.

The most frequently reported vaccines for children aged <10 and 10–19 years were the *Haemophilus influenzae* type b conjugate vaccine (19.2%) and the recombinant adsorbed bivalent human papillomavirus-like particle vaccine (derived from *Trichoplusia ni* cells) (65.7%) (Table 2). The number of reports regarding the pneumococcal 13-valent conjugate vaccine (diphtheria CRM197 protein) increased, and it became the most commonly reported vaccine in recent years for children aged <10 (Figure 2). For children aged <10 and 10–19 years, from 2004–2009, the influenza hemagglutinin vaccine was the most frequently reported. After 2010, reports regarding the recombinant adsorbed bivalent human papillomavirus-like particle vaccine increased, and it was the most frequently reported vaccine in recent years (Figure 2). Among AEFI reports with death as an outcome for children aged <10, the most frequently reported vaccines were the *Haemophilus influenzae* type b conjugate vaccine (29.5%) and, for children aged 10–19 years, the recombinant adsorbed bivalent human papillomavirus-like particle vaccine (50.0%).

### 3.4. Ten Most Frequently Reported Reactions

**Figure 3:**
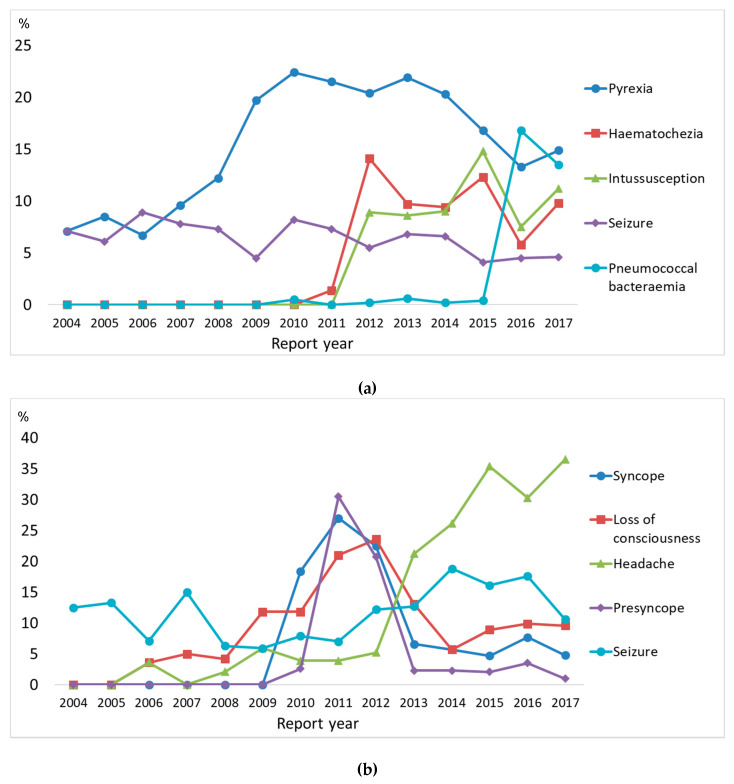
Time trend of five most frequently reported adverse reactions according to age group. (**a**) Children <10 years old. (**b**) Children 10–19 years old.

The most frequently reported reactions described in PT for children aged <10 and 10–19 years were pyrexia (9.9%) and syncope and a loss of consciousness (3.8%), respectively (Table 2). Among AEFI reports with death as an outcome for children aged <10 and 10–19 years, the most frequently reported reactions were cardiorespiratory arrest (14.3% and 6.7%, respectively). For children aged <10, pyrexia has frequently been reported in each reporting year. Syncope and a loss of consciousness have increased since 2010 for children aged <10 and 10–19 years (Figure 3).

### 3.5. Ten Most Frequently Reported Vaccine–Reaction Pairs

**Figure 4:**
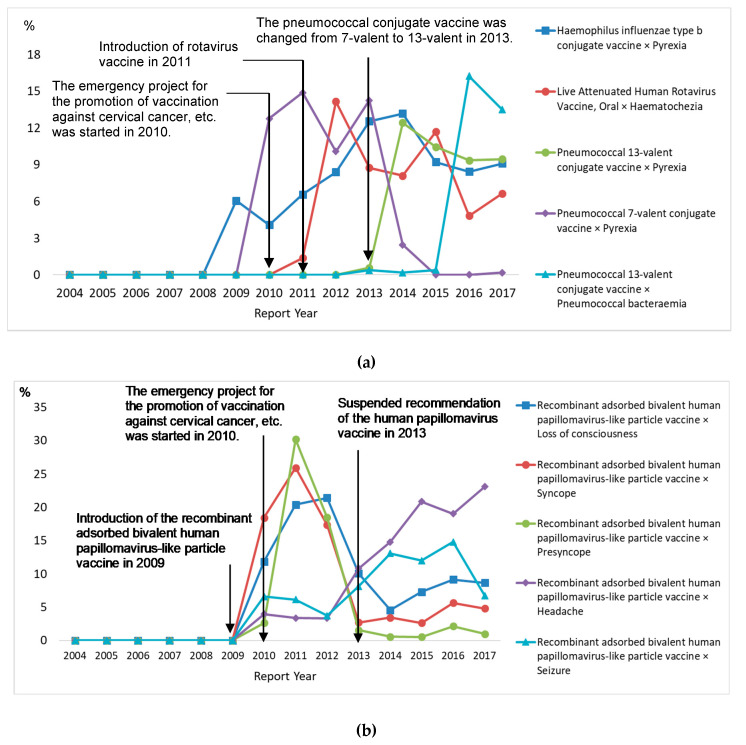
Time trend of five frequently reported vaccines–reaction pair according to age group. (**a**) Children <10 years old. (**b**) Children 10–19 years old.

The most frequently reported vaccine–reaction pair was the *Haemophilus influenzae* type b conjugate vaccine with pyrexia (2.6%) among children aged <10 and the recombinant adsorbed bivalent human papillomavirus-like particle vaccine (derived from *Trichoplusia ni* cells) with a loss of consciousness (3.4%) among children aged 10–19 years (Table 3). Pyrexia related to the *Haemophilus influenzae* type b conjugate vaccine and the pneumococcal conjugate vaccine has increased since 2009, and incidences of live attenuated human rotavirus vaccine with hematochezia reactions have increased since 2011 among children aged <10. Various reactions, including a loss of consciousness, syncope, and presyncope, in relation to the recombinant adsorbed bivalent human papillomavirus-like particle vaccine were reported in 2011 and had declined and levelled off among children aged 10–19 years (Figure 4). Among AEFI reports with death as an outcome for children aged <10, the most frequently reported drug–reaction pairs were “the *Haemophilus influenzae* type b conjugate vaccine and cardiorespiratory arrest” (4.3%) and, for children aged 10–19 years, “the recombinant adsorbed bivalent human papillomavirus-like particle vaccine and ventricular fibrillation” and “the recombinant adsorbed bivalent human papillomavirus-like particle vaccine and respiratory failure” (6.7%) (Table 4).

## 4. Discussion

It is necessary to grasp the rate of AEFI for the assessment of vaccine safety. However, in Japan, the history of individual vaccinations has not been recorded in a database. It was difficult to estimate the number of children that received the vaccines because vaccinations are not covered by health insurance and they do not remain as health insurance claims data, and, even for vaccinations provided free by public funds, the information is recorded with various forms by each local government and rarely published. Therefore, there is no way to estimate the number of shots delivered or the number of children who received the shots. Furthermore, the reports included in the JADER are part of the AEFI reports registered by the PMDA, so the population denominator cannot provide an indication as to how well the system is operating. In the previous study, the reporting rate per 1,000,000 population was low in Japan [12] and not in line with other developed countries. The low rates of spontaneous reporting might be considered indicative of a not well-functioning system in Japan now. MHLW provided the basic plan for vaccination in 2014. In this plan, the establishment of the database that can grasp the rate of vaccination, the digitization of AEFI reports, and the utilization of the national receipt database and various statistics were considered to promote measures for vaccination comprehensively and systematically [13]. To realize this basic plan, it is necessary to understand the actual situation of pharmacovigilance activity regarding vaccination in Japan and clarify the issues regarding vaccination. However, there are no prior studies of AEFI reports among Japanese pediatric patients in the literature. Accordingly, the present study provides an overview of AEFI in Japanese children from 2004–2017.

Our analysis found that although the causality between the suspected vaccine and death as an outcome has not been analyzed, the proportion of AEFI reports with death as an outcome for children aged <10 was higher than that found in other countries (e.g., 0.71% in the UK, 0.04% in Malaysia, and 0.28% in Spain) [4,5,6]. One explanation for the high proportion of AEFI reporting with death as an outcome could be that the JADER is a “spontaneous” ADR database, comprising serious ADR reports selected by the PMDA, while databases in other countries include nonserious ADR reports. The proportion of AEFI reporting with death may also reflect differences in the medical environment and attitudes toward spontaneous reporting in each country [14]. Hesitancy concerning vaccines because of vaccine safety skepticism is prevalent in Japan [7,8], which might lead to AEFI with death, as outcomes being reported more often regardless of the causal relationship between vaccination and death. For example, for “the *Haemophilus influenzae* type b conjugate vaccine and cardio-respiratory arrest” pair, according to Pharmaceuticals and Medical Devices Safety Information published by MHLW [15], infections, underlying heart disease, and sudden infant death syndrome accounted for most of the causes of deaths, and there was no clear causality between the vaccination and death.

Our study also suggests that information regarding age is essential in discussions about AEFI, especially in relation to children. The characteristics of AEFI reports varied considerably by pediatric patient age in previous reports [5]. Therefore, we need to consider pediatric AEFI reports with more detailed age classifications because the potential risk of serious AEFI and the types of vaccines vary with age. The timing of the administration of each vaccine has been recommended in detail by the Japan Pediatric Society. According to the immunization schedule [16], the adsorbed diphtheria-purified pertussis-tetanus-inactivated polio combined vaccine and pneumococcal conjugate vaccine should be administered four times for children less than 2 years old, and the recombinant adsorbed bivalent human papillomavirus-like particle vaccine is administered to children aged 11 to 16 years. In the present study, the ten most frequently reported vaccines appeared to be relatively different between the two age groups. However, we could not obtain age information as a continuous variable, as, although age information should have been reported as a continuous variable in the original ADR reports, the JADER only includes age information as a categorical variable because of privacy considerations. To promote the safety evaluation of vaccines in children, age information as a continuous variable should be disclosed in the JADER, especially for pediatric ADR reports.

The number of suspected vaccines per AEFI report and the number of AEFI reports have been increasing. The timing of the increase in the number of AEFI reports coincided with the introduction of a new vaccine, which is the so-called Weber effect. Moreover, in November 2010, the vaccination of the *Haemophilus influenzae* type b vaccines, the pediatric pneumococcal conjugate vaccine, and the human papillomavirus vaccine for cervical cancer prevention were promoted publicly in Japan [17]. The spiked number of AEFI reports regarding the recombinant adsorbed bivalent human papillomavirus-like particle vaccine in the JADER in 2011 might be caused by the Weber effect and this promotion project. Furthermore, the number of AEFI reports regarding syncope might have been increased because the guideline of this promotion project stated that syncope might occur as a vasovagal reflex after the vaccination of the human papillomavirus vaccine and it was desirable to observe the condition of the children who received the vaccine [17]. This was the reason that the percentage of AEFI reports corresponding to girls was approximately 90% among children aged 10–19 years. Moreover, in 2011, the Japan Pediatric Society announced its recommendation of an immunization schedule and the concept of simultaneous administration of multiple vaccines in clinical settings [18]. The Weber effect was sometimes cited as limitations to the usefulness of a spontaneous reporting system. Although it was a study excluding vaccine reports, the United States Food and Drug Administration’s (FDA) Adverse Event Reporting System (FAERS), which is widely used to support post-marketing safety surveillance programs in the US, could have excluded the weber effect by the improvement of both volume and quality of ADR reporting [19]. To exclude them, the JADER needs the large increases in the volume of AEFI reports, as well as a concerted effort to increase awareness regarding the utility of post-marketing ADR reporting.

In June 2013, the Ministry of Health, Labour and Welfare suspended proactive recommendations for the human papillomavirus vaccine. The proactive recommendation has still been suspended, although the causal relationship between the human papillomavirus vaccine and various adverse reactions has not been clarified in the Nagoya study [20]. Japan is unable to assess vaccine concerns, such as the human papillomavirus vaccine. However, in 2017, the Japan Expert Council on Promotion of Vaccination—a body of 17 academic societies from a broad range of fields, including infectious disease, pediatrics, obstetrics and gynecology, respiratory illness, travel health, and vaccinology—published a statement recommending renewed proactive support for the widespread use of the human papillomavirus vaccine [21] and the Japan Society of Obstetrics and Gynecology submitted a request form regarding the proactive recommendations for the human papillomavirus vaccine in November 2019. There is much debate about the human papillomavirus vaccine in Japan. Thus, we contend that the JADER should be interpreted with caution because an event occurring after vaccination is not necessarily caused by the vaccination, and the quality of the ADR reports in the JADER has varied historically [22,23].

The amendment of the Preventive Vaccination Law in 2013 also may affect the number of AEFI reports. Before the amendment, adverse events following vaccination were not necessarily reported to the PMDA, so not all serious cases reported as AEFI were entered into the JADER database. However, all the serious cases reported will have been entered into the JADER following the amendment in 2013; therefore, the relative number of vaccine reports might have increased accordingly. The JADER is the only way to monitor the reporting of AEFI, as the history of individual vaccinations has not otherwise been recorded in Japan. Therefore, the present study’s attempt to clarify the characteristics of AEFI in the JADER should prove useful for the identification of issues that should be addressed in Japan. “Recommendation on the Reporting System of Adverse Reactions to Vaccines and the Development of Infrastructure related to Vaccine Risk Management in Japan” was published in 2015 [24] and the preparation of the revised version are currently on going to call for urgent attention on the country’s vaccine safety surveillance capacity and recommend the implementation of systems for signal verification.

As the JADER is a spontaneous ADR reporting database, it has multiple limitations, including regarding the reporting of temporal associations, the unconfirmed diagnoses, a lack of denominator of the user, and an unbiased comparison of groups [1]. There is no way to estimate the number of shots delivered or the number of children who received the shots, especially because of the lack of a denominator. In the JADER, detailed information on the source of spontaneous ADR reports was not revealed. Therefore, there remains the possibility of duplicated reports, whereby one case might be reported multiple times. This possibility cannot be completely excluded because there are no identifiers for the same case. The identification and elimination of duplicates from an analysis are advantageous and important for the correct interpretation of the data. Moreover, it was not possible to analyze the situation by the detailed age because the JADER only included age information as a categorical variable, such as children aged <10 and 10–19 years. Because of these limitations, causality between vaccines and adverse reactions from the JADER cannot be evaluated. However, the database enables the early detection of signals that can then be investigated more deeply [25]. To increase the availability and value of the JADER, age information as a continuous variable should be disclosed, especially in pediatric AEFI reports.

## 5. Conclusions

The present study clarified the trends and characteristics of AEFI reports in Japanese children using the JADER. It is necessary to consider the weber effects to understand the trend and characteristics of AEFI reporting because pharmacovigilance activity regarding vaccination is not sufficient in Japan.

## Figures and Tables

**Table 1:** Characteristics of AEFI reports according to age group.

Characteristics	Age Group		*p*
	<10 Years ^a^ *n* (%)	10–19 Years ^b^ *n* (%)	
**Sex**			
Boy	2272 (53.7)	208 (10.1)	<0.0001
Girl	1801 (42.6)	1838 (89.7)	
Unknown	157 (3.7)	4 (0.2)	
**The type of report sender**			
Pharmaceutical company	4155 (98.2)	2047 (99.9)	<0.0001
Healthcare facility	75 (1.8)	3 (0.1)	
**The qualifications of the reporter**			
Doctor	3548 (73.7)	1807 (58.0)	<0.0001
Pharmacist	157 (3.3)	99 (3.2)	
Healthcare professional	358 (7.4)	201 (6.5)	
Consumer	335 (7.0)	919 (29.5)	
Lawyer	0 (0.0)	21 (0.7)	
Unknown	414 (8.6)	67 (2.2)	
Total ^c^	4812 (100)	3114 (100)	-
**No. of suspected vaccines per AEFI report**			
1	2673 (63.2)	2028 (98.9)	<0.0001
2	554 (13.1)	22 (1.1)	
3	485 (11.5)	0 (0.0)	
4	347 (8.2)	0 (0.0)	
≥5	171 (4.0)	0 (0.0)	
Total	4230 (100)	2050 (100)	-
**Outcomes associated with AEFI report**			
Cured	3934 (52.6)	3734 (44.3)	<0.0001
Recovering	1585 (21.2)	1024 (12.2)	
Did not recover	276 (3.7)	1776 (21.1)	
Recovering with sequelae	91 (1.2)	51 (0.6)	
Death	321 (4.3)	15 (0.2)	
Unknown	1272 (17.0)	1826 (21.7)	
Total ^d^	7479 (100)	8426 (100)	-

Abbreviation: AEFI, adverse event following immunization. ^a^ *n* = 4230 (67.4%). ^b^ *n* = 2050 (32.6%). ^c^ If there were multiple reporters in a single report, each was counted. ^d^ If there were multiple AEFIs in a single report, each was counted.

**Table 2:** Ten most frequently reported vaccines and reactions according to age group.

**<10 Years**	***n* (%)**
**Vaccine Name ^a^**	
*Haemophilus influenzae* type b conjugate vaccine (monovalent)	1438 (19.2)
Pneumococcal 13-valent conjugate vaccine (diphtheria CRM197 protein)	1163 (15.5)
Live attenuated human rotavirus vaccine, oral (monovalent)	775 (10.3)
Pneumococcal 7-valent conjugate vaccine (diphtheria CRM197 protein)	616 (8.2)
Adsorbed diphtheria-purified pertussis-tetanus-inactivated polio (Sabin strain) combined vaccine	521 (7.0)
Influenza hemagglutinin vaccine	517 (6.9)
Recombinant adsorbed hepatitis B vaccine (yeast derived)	412 (5.5)
Rotavirus vaccine, live, oral, pentavalent	376 (5.0)
Freeze-dried live attenuated mumps virus vaccine	313 (4.2)
Freeze-dried bacillus Calmette–Guérin vaccine	267 (3.6)
**Adverse reactions ^b^**	
Pyrexia	731 (9.9)
Hematochezia	317 (4.3)
Intussusception	314 (4.3)
Seizure	245 (3.3)
Pneumococcal bacteremia	193 (2.6)
Immune thrombocytopenic purpura	190 (2.6)
Anaphylactic reaction	188 (2.6)
Meningitis aseptic	179 (2.4)
Vomiting	178 (2.4)
Febrile convulsion	173 (2.3)
**10–19 years**	***n* (%)**
**Vaccine name ^c^**	
Recombinant adsorbed bivalent human papillomavirus-like particle vaccine (derived from *Trichoplusia ni* cells)	1361 (65.7)
Recombinant adsorbed quadrivalent human papillomavirus virus-like particle vaccine (yeast origin)	295 (14.2)
Influenza hemagglutinin vaccine	164 (7.9)
Freeze-dried live attenuated mumps virus vaccine	90 (4.3)
Freeze-dried live attenuated measles and rubella combined vaccine	50 (2.4)
Freeze-dried, cell culture-derived Japanese encephalitis vaccine	31 (1.5)
Adsorbed diphtheria–tetanus combined toxoid	30 (1.4)
Japanese encephalitis vaccine (not otherwise specified)	11 (0.5)
Recombinant adsorbed hepatitis B vaccine (yeast derived)	10 (0.5)
Dried live attenuated measles vaccine	9 (0.4)
**Adverse reactions ^d^**	
Syncope	312 (3.8)
Loss of consciousness	308 (3.8)
Headache	297 (3.6)
Presyncope	287 (3.5)
Seizure	233 (2.8)
Malaise	196 (2.4)
Pyrexia	193 (2.4)
Pain	178 (2.2)
Hypoesthesia	148 (1.8)
Dizziness	146 (1.8)

Notes: The terms were described in Japanese (MedDRA^®^/J Version 21.0). ^a^ *n* = 7494. ^b^ *n* = 7363. ^c^ *n* = 2072. ^d^ *n* = 8176.

**Table 3:** Ten most frequently reported vaccine–reaction pairs according to age group.

Generic Name	Adverse Reactions	*n* (%)
<10 Years		
*Haemophilus influenzae* type b conjugate vaccine	Pyrexia	357 (2.6)
Live attenuated human rotavirus vaccine, oral	Hematochezia	278 (2.1)
Pneumococcal 13-valent conjugate vaccine (diphtheria CRM197 protein)	Pyrexia	234 (1.7)
Pneumococcal 7-valent conjugate vaccine (diphtheria CRM197 protein)	Pyrexia	199 (1.5)
Pneumococcal 13-valent conjugate vaccine (diphtheria CRM197 protein)	Pneumococcal bacteremia	186 (1.4)
Live attenuated human rotavirus vaccine, oral	Intussusception	182 (1.3)
Freeze-dried live attenuated mumps virus vaccine	Meningitis aseptic	162 (1.2)
Live attenuated human rotavirus vaccine, oral	Pyrexia	161 (1.2)
Rotavirus vaccine, live, oral, pentavalent	Intussusception	130 (1.0)
Adsorbed diphtheria-purified pertussis-tetanus-inactivated polio (Sabin strain) combined vaccine	Pyrexia	129 (1.0)
**10–19 years**		
Recombinant adsorbed bivalent human papillomavirus-like particle vaccine (derived from *Trichoplusia ni* cells)	Loss of consciousness	277 (3.4)
	Syncope	270 (3.3)
	Presyncope	269 (3.3)
	Headache	180 (2.2)
	Seizure	152 (1.8)
	Depressed level of consciousness	135 (1.6)
	Malaise	132 (1.6)
	Pyrexia	122 (1.5)
	Pain	120 (1.5)
	Arthralgia	108 (1.3)

**Table 4:** Ten most frequently reported vaccine–reaction pairs in AEFI reports with the death as an outcome according to age group.

Generic Name	Adverse Reactions	*n* (%)
<10 years		
Haemophilus influenzae type b conjugate vaccine	Cardio-respiratory arrest	51 (4.3)
Haemophilus influenzae type b conjugate vaccine	Sudden infant death syndrome	39 (3.3)
Pneumococcal 13-valent conjugate vaccine (diphtheria CRM197 protein)	Cardio-respiratory arrest	30 (2.5)
Haemophilus influenzae type b conjugate vaccine	Death	29 (2.4)
Adsorbed diphtheria-purified pertussis-tetanus-inactivated polio (Sabin strain) combined vaccine	Cardio-respiratory arrest	23 (1.9)
Pneumococcal 13-valent conjugate vaccine (diphtheria CRM197 protein)	Sudden infant death syndrome	21 (1.8)
Haemophilus influenzae type b conjugate vaccine	Respiratory arrest	21(1.8)
Pneumococcal 13-valent conjugate vaccine (diphtheria CRM197 protein)	Death	20 (1.7)
Pneumococcal 7-valent conjugate vaccine (diphtheria CRM197 protein)	Cardio-respiratory arrest	19 (1.6)
Adsorbed diphtheria-purified pertussis-tetanus-inactivated polio (Sabin strain) combined vaccine	Sudden infant death syndrome	18 (1.5)
Pneumococcal 13-valent conjugate vaccine (diphtheria CRM197 protein)	Sudden infant death syndrome	18 (1.5)
**10–19 years**		
Recombinant adsorbed bivalent human papillomavirus-like particle vaccine (derived from *Trichoplusia ni* cells)	Ventricular fibrillation	2 (6.7)
	Respiratory failure	2 (6.7)
	Altered state of consciousness, Bulbar palsy, Ventricular tachycardia, Amyotrophic lateral sclerosis, Cardio-respiratory arrest, Bundle branch block left, Musculoskeletal stiffness, Tension headache, Sinus arrhythmia, Electroencephalogram abnormal, Osteosarcoma, Loss of consciousness, Ventricular arrhythmia, Dyspnoea, Completed suicide, Arrhythmia supraventricular, Respiratory arrest, Muscular weakness, Seizure, Ventricular extrasystoles	1 (3.3) for each adverse reaction
Influenza HA vaccine	Myocarditis	1 (3.3)
Freeze-dried, Cell Culture-derived Japanese. Encephalitis Vaccine	Syncope	1 (3.3)
Freeze-dried live attenuated measles and rubella combined vaccine	Cardio-respiratory arrest	1 (3.3)
Pneumococcal vaccine	Pneumococcal infection, Post procedural infection, Sudden death	1 (3.3) for each adverse reaction
